# Integrating GLP-1 Receptor Agonists into Modern Stroke Prevention: Evidence, Mechanisms, and Clinical Consideration—A Narrative Review

**DOI:** 10.3390/biomedicines14040743

**Published:** 2026-03-24

**Authors:** Shayan Khan, William Herbst, Farbod Zahedi Tajrishi, Sonali Notani, Alexander Knight, Zina Jamil, Keith C. Ferdinand

**Affiliations:** 1 Department of Neurology, Tulane University School of Medicine, 1430 Tulane Avenue, New Orleans, LA 70112, USA; skhan19@tulane.edu; 2 Department of Medicine, Tulane University School of Medicine, 1430 Tulane Avenue, New Orleans, LA 70112, USA; wherbst@tulane.edu (W.H.); snotani@tulane.edu (S.N.); 3 Department of Internal Medicine, Tulane University School of Medicine, 1430 Tulane Avenue, New Orleans, LA 70112, USA; fzaheditajrishi@tulane.edu (F.Z.T.); gknight2@tulane.edu (A.K.); 4 Department of Trauma and Orthopedics, Craigavon Area Hospital, 68 Lurgen Rd, Portadown, BT63 5QQ Craigavon, Ireland; jamil_zina@yahoo.com; 5 Department of Cardiology, Tulane University School of Medicine, 1430 Tulane Avenue, New Orleans, LA 70112, USA

**Keywords:** acute ischemic stroke, type 2 diabetes mellitus, GLP-1 receptor agonist, neurovascular protection, inflammation, endothelial dysfunction, cardiovascular outcomes, cerebrovascular disease

## Abstract

Stroke remains a major cause of morbidity and mortality worldwide. Although reperfusion therapies and secondary prevention have advanced, the global stroke burden continues to rise, driven by increasing rates of hypertension and diabetes mellitus. Type 2 diabetes (T2DM) increases the risk of acute ischemic stroke (AIS) through mechanisms involving chronic hyperglycemia, endothelial dysfunction, inflammation, and accelerated atherogenesis. In recent years, glucagon-like peptide-1 receptor agonists (GLP-1RAs) have emerged as promising agents for cardiovascular and cerebrovascular risk reduction in patients with T2DM. Beyond their glucose-lowering properties, GLP-1RAs improve blood pressure regulation and lipid metabolism, as mentioned in the 2025 AHA Journal guidelines for the prevention, detection, evaluation, and management of high blood pressure in adults. Emerging preclinical and clinical evidence indicates that GLP-1RAs also provide direct neurovascular protection by stabilizing the blood–brain barrier, modulating neuroinflammation, and promoting neuronal survival. These mechanisms may reduce ischemic injury, improve recovery after stroke, and protect against cognitive decline. Major cardiovascular outcome trials have demonstrated significant reductions in major adverse cardiovascular events and, to a lesser degree, non-fatal stroke among patients receiving GLP-1RAs. This narrative review evaluates current evidence on the neurovascular, cardiometabolic, and anti-inflammatory actions of GLP-1RAs and their potential role in mitigating stroke risk and promoting cerebrovascular health. Additionally, it highlights gaps in the literature, explores clinical and guideline implications, and outlines future directions for integrating GLP-1RA therapy into comprehensive stroke prevention and recovery strategies.

## 1. Introduction

### 1.1. Clinical Background and Rationale

In the United States, 17.5% of deaths from cardiovascular disease in 2022 were due to stroke. Both type 1 and type 2 diabetes (T2DM) have a substantially increased risk of acute ischemic stroke (AIS), approximately 1.5–2 times higher than the general population. Since the introduction of exenatide, the world’s first glucagon-like-peptide 1 receptor agonists (GLP-1RA) developed in 1995 and approved for marketing in 2005, GLP-1RA products have emerged as a promising therapeutic agent for cardiovascular risk reduction, particularly in patients with T2DM and elevated stroke risk [1].

The American Heart Association (AHA) and American Stroke Association (ASA) guidelines for comprehensive stroke care as well as the 2025 AHA journal guidelines for the prevention, detection, evaluation, and management of high blood pressure in adults emphasize comprehensive risk factor management for vascular prevention in patients with diabetes. This includes glycemic control, blood pressure management, lipid optimization, antiplatelet therapy, and lifestyle interventions [2]. The relative risk of stroke in patients with T2DM is dependent on stroke subtypes. In the review, we will classify strokes based on the Trial of ORG 10172 in Acute Stroke Treatment (TOAST) stroke subtype classification system [3]. Patients with T2DM tend to have an increased risk of large artery atherosclerosis, cerebral small vessel disease, and cardiac embolic strokes. However, the majority of patients with T2DM suffer from small vessel diseases [4]. In this narrative review, we will explore the benefits of GLP-1RA regarding their current clinical use in cardiometabolic disease prevention with a focus on stroke. Major cardiovascular outcome trials such as LEADER (Liraglutide Effect and Action in Diabetes: Evaluation of Cardiovascular Outcome Results), SUSTAIN-6 (Trial to Evaluate Cardiovascular and Other Long-term Outcomes with Semaglutide), and REWIND (Researching Cardiovascular Events with a Weekly Incretin in Diabetes) have demonstrated significant reductions in major adverse cardiovascular events, including non-fatal stroke, among patients receiving GLP-1RAs.

### 1.2. Methodology

A narrative review was carried out following the Scale for the assessment of Narrative Review Articles (SANRA) guidelines. Electronic databases such as Pubmed, Google Scholar and Medline were searched for relevant published articles between the years 2022 and 2025 that addressed the cardiovascular, cerebrovascular, metabolic, or neuroprotective effects of GLP-1 receptor agonists. Titles and abstracts were screened for relevance; the full text was analyzed when relevant, and reference lists of included studies were manually searched to identify additional articles.

A combination of keywords such as “acute ischemic stroke; type 2 diabetes mellitus; GLP-1 receptor agonist; neurovascular protection; inflammation; endothelial dysfunction; cardiovascular outcomes; and cerebrovascular disease” were searched. Observational studies, cohort studies, and case series were considered with the prioritization of large randomized controlled trials. Articles were excluded that were not available in English, or did not directly address the topic of interest. Relevant data and key findings were extracted; the literature was then organized to summarize the most updated evidence and identify gaps in the existing research. Bio-render (Science Suite Inc., Toronto, ON, Canada) was used to create graphical figures, appropriate copyright was obtained and reference management was performed using Zotero (Corporation for Digital Scholarship, Falls Church, VA, USA). All figures and tables in this manuscript are non-published and original.

As it was not conducted as a formal systematic review, the limitation encountered was a lack of structured risk of bias assessment; thus, included studies were intended to be representative rather than exhaustive.

## 2. Diabetes, Cerebrovascular Injury, and the Potential Role of GLP-1RAs

Hyperglycemia is the main driving factor in T2DM and is defined as a blood glucose level of 6 mmol (180). It has been observed in up to two thirds of all ischemic stroke subtypes on admission and at least 50% in each subtype [5]. Patients with hyperglycemia compared to patients without show expanded infarct sizes, resulting in adverse effects on tissue outcomes [4,6]. The evolution of an acute infarct causes the release of glutamate, which results in spreading depression that is theorized to propagate necrosis of the penumbra [7]. Although hyperglycemia does not directly trigger spreading depression, elevated glucose may alter gene expression in at risk neurons (penumbra). Furthermore, the blood–brain barrier (BBB) is vulnerable to hyperglycemia due to increased lactate production and release of free radicals [8].

Higher fasting blood glucose (FBG) on admission independently predicts unfavorable outcomes and increased mortality at 90 days in diabetic patients with acute ischemic stroke, despite adjusting for confounders, as measured by the National Institutes of Health Stroke Scale and the modified Rankin Scale (mRS). Similarly, glycemic variability during hospitalization is associated with worse 3-month functional outcomes (mRS 3–6) in diabetic patients, with higher glucose ranges correlating with increased risk of poor outcome [9,10]. Hyperglycemia has been linked with hemorrhagic events in post-thrombolytic treatment in multiple thrombolysis trials, leading to increased morbidity in this subgroup [5].

**Figure 1:**
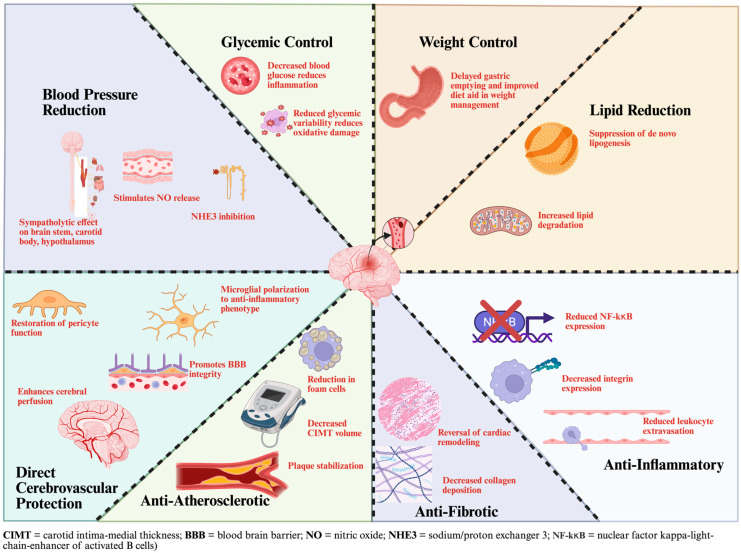
Protective effects of GLP-1RAs against stroke [13,14,15,16,17]. Created in BioRender. Herbst, W. (2026) https://www.biorender.com/ii2uazi (accessed on 20 March 2025).

Dulaglutide demonstrated consistent cardiovascular benefit in Researching Cardiovascular Events with a Weekly Incretin in Diabetes (REWIND) among participants with and without established cardiovascular disease, with no evidence of heterogeneity between subgroups. This consistency is noteworthy given that only 31.5% of REWIND participants had pre-existing cardiovascular disease, making it the only GLP-1 receptor agonist trial predominantly enrolling a primary prevention population. The uniformity of effect across subgroups supported dulaglutide’s FDA approval for cardiovascular risk reduction in adults with type 2 diabetes, irrespective of established atherosclerotic cardiovascular disease. A subsequent meta-analysis incorporating REWIND similarly found no significant interaction between baseline cardiovascular disease status and treatment effect, contrasting earlier analyses of GLP-1 RAs that had suggested benefit primarily in secondary prevention populations. These findings were further affirmed in the Liraglutide Effect and Action in Diabetes: Evaluation of Cardiovascular Outcome Results (LEADER) trial, which compared liraglutide to placebo in patients with T2DM. In the population groups selected, 81% of the patient population had prior cardiovascular disease. Patients on liraglutide had a lower likelihood of cardiovascular events compared to the placebo group [11,12]. The Potential protective effects of GLP-1RAs against stroke have been summarized in Figure 1 [13,14,15,16,17] and further expanded on in the rest of the review.

## 3. Potential Mechanisms of Stroke Risk Reduction with GLP-1RAs

### 3.1. Glycemic Control

Elevated HbA1c is strongly associated with stroke [2,18]. Each 1-unit rise in HbA1c increases risk of macrovascular events (myocardial infarction [MI], stroke, peripheral artery disease) by ~18%, and maintaining HbA1c < 7% reduces CVD risk by ~37% over 11 years [19]. GLP-1RAs consistently lower HbA1c across large randomized controlled trials and are associated with improved cardiovascular outcomes [20]. Both acute and chronic hyperglycemia drive oxidative stress and endothelial dysfunction, accelerating atherogenesis and impairing cerebrovascular structure and function. At the vessel wall, high glucose increases reactive oxygen species, activates the Nuclear Factor kappa light chain-enhancer of activated B cells (NF-κB), and promotes advanced glycation end-product receptors for advanced glycation end-product (AGE–RAGE) signaling, which upregulates adhesion molecules and inflammatory cytokines, all of which are core steps in plaque formation and instability [21]. Hyperglycemia also amplifies matrix metalloproteinase-9 (MMP-9) activity and disrupts the BBB, leading to vasogenic edema, leukocyte infiltration, and secondary neuronal injury after ischemia [22]. These pathophysiologic effects may explain why higher glucose at baseline and during acute stroke is linked to larger infarcts, worse outcomes, and greater mortality [4,6,23,24].

GLP-1RAs control blood glucose through multiple mechanisms related to mimicking the hypoglycemic effects of incretin, which increases insulin secretion, suppresses glucagon release, inhibits appetite, and slows gastric emptying. By slowing gastric emptying, they attenuate post-prandial glucose spikes, which has a moderating effect on long- and short-term blood glucose levels [25,26,27]. Central satiety signaling and appetite suppression are induced by the insulin and glucagon effects, which lead to reduced meal size and caloric load, further smoothing postprandial glucose profiles [4,6,28,29].

Beyond HbA1c, glycemic variability is independently associated with vascular outcomes. In the Intensive Glucose Lowering and Its Effects on Vascular Events and Death According to Age at Diagnosis and Duration of Diabetes (ADVANCE) trial, visit-to-visit variability in HbA1c and fasting glucose correlated with higher rates of cardiovascular events and mortality [24]. In the Efficacy and Safety of Degludec versus Glargine in Type 2 Diabetes (DEVOTE) trial, doubling of day-to-day fasting glucose variability was linked to major adverse cardiovascular events and all-cause mortality [30]. At the cellular level, intermittent high glucose drives more oxidative stress and apoptosis in endothelial cells compared with constant hyperglycemia [31]. GLP-1RAs consistently reduce glycemic variability. A meta-analysis of 16 trials confirmed that GLP-1RAs significantly reduce multiple indices of glycemic variability, including mean amplitude of glycemic excursions, coefficient of variation, standard deviation, and time above range [32,33]. This is mainly achieved via their incretin-associated properties explained above, which maintain blood glucose within physiological levels and prevent both hypoglycemic and hyperglycemic episodes.

### 3.2. Blood Pressure Control and Modification of Lipid Metabolism

Weight reduction remains a cornerstone of blood pressure (BP) management. Current 1A recommendations from the 2025 AHA/American College of Cardiology guidelines, as well as the ASA, recommend treating most neurologically stable patients with a history of ischemic stroke or TIA to achieve a blood pressure goal of less than 130/80 mm Hg for optimal reduction in recurrent stroke and major vascular events. It is also recommended that adults with overweight or obesity should pursue weight loss of at least 5% of body weight to prevent or treat elevated BP and hypertension, with evidence showing an approximate 1/1 mm Hg (systolic/diastolic) BP reduction for each kilogram of weight lost. Greater degrees of weight loss produce proportionally larger BP reductions in individuals with and without hypertension.

GLP-1 receptors are expressed in the central nervous system, vasculature, and kidneys, where GLP-1R activation promotes blood pressure reduction through multiple mechanisms. Activation of cardiac GLP-1R activation increases atrial natriuretic peptide secretion, leading to natriuresis and vasodilation [34]. Mouse models provide strong evidence that GLP-1RAs exert a direct sympatholytic effect through receptors on the hypothalamus, carotid body, and brainstem. In addition to decreasing urinary norepinephrine in hypertensive mice, an index of renal sympathetic activity, they have been shown to act directly on the proximal tubule to inhibit the sodium/hydrogen exchanger 3 (NHE3), further increasing sodium excretion and reducing blood pressure [35,36]. Antioxidant and increased endothelial nitric oxide synthase activity are additional mechanisms for the antihypertensive effects of GLP-1RAs.

The appetite-modulating effects of these agents may extend to dietary quality. Emerging evidence from animal studies suggests that GLP-1RAs may reduce alcohol consumption and cigarette use [37,38]. Such behavioral modifications, combined with the direct pharmacological effects on natriuresis, may synergistically contribute to blood pressure reduction.

Accumulating evidence suggests GLP-1RAs directly affect lipid metabolism and blood pressure independent of their appetite-suppressing properties. GLP-1RAs directly modulate hepatic lipid metabolism by suppressing de novo lipogenesis via Adenosine monophosphate-activated protein kinase (AMPK) activation and downregulation of lipogenic genes (peroxisome proliferator-activated receptor-γ [PPARγ]) and sterol regulatory element-binding protein 1c [SREBP-1c], acyl-CoA synthase long chain family member 1 (ACSL1), and enhancing fatty acid oxidation [39]. They promote reverse cholesterol transport and decreased cholesterol synthesis through both ATP-binding cassette transporter (ABCA1) upregulation and 3-Hydroxy-3-methylglutaryl-coenzyme A (HMG-CoA) reductase inhibition, also inducing autophagy-mediated lipid degradation [40,41,42,43,44].

Higher serum LDL-C levels have been shown to be linearly associated with an elevated risk of incidental diabetes and are known to be linked with increased risk of AIS [45]. Exploratory findings from STEP 1 and STEP 4 trials show that semaglutide produces meaningful reductions in LDL-C and non-HDL cholesterol, triglycerides, and markers of insulin resistance, with the magnitude of improvement correlating closely with the degree of weight loss patients experienced. However, once the medication is stopped, these cardiovascular benefits do not continue with reversal of all gains made in the patient population, highlighting the importance of continued therapy to maintain benefit [46,47]. Consistent compliance with GLP-1RAs leads to a reduction in systolic blood pressure by 2 to 3 mm Hg in subjects with hypertension [48,49,50,51]. However, further investment in clinical trials will be needed to further evaluate if there is any further direct improvement in blood pressure.

The blood pressure-lowering effect of GLP-1RAs is an additive to conventional antihypertensive therapy, being generally well tolerated, and should be used as part of the overall cardiometabolic risk reductions; however, GLP-1RAs are not a replacement for a comprehensive antihypertensive regimen [52].

### 3.3. Anti-Inflammatory, Anti-Fibrotic, and Anti-Atherosclerotic Properties

Independent of glycemic and metabolic control, GLP-1RAs exert broad anti-inflammatory, antioxidative, and anti-fibrotic effects that may contribute directly to stroke protection. Since the late 1990s, atherosclerosis has been recognized as primarily an inflammatory condition [53]. Every step in the atherosclerotic process, from the initial binding of leukocytes to damaged endothelium to the structural weakness that causes atheroma to rupture and thrombose, is associated with pro-inflammatory markers and chemokines. Blood levels of these markers, such as tumor necrosis factor- alpha (TNF-a), c-reactive protein and interleukin-6 predict outcomes of patients with acute coronary syndrome independent of myocardial damage [54]. The Anti-inflammatory Therapy with Canakinumab for Atherosclerotic Disease (CANTOS) trial demonstrated that targeting inflammation itself may lower vascular risk: in over 10,000 post-MI patients, IL-1β inhibition with canakinumab was associated with decreased major cardiovascular events by 15%, independent of lipid levels [55]. This supports inflammation as a crucial contributor to atherosclerotic disease.

The American College of Cardiology (ACC) released a statement identifying high-sensitivity C-reactive protein (hs-CRP) as a clinically actionable vascular risk factor as significant as LDL-C or systolic blood pressure [56]. In the STEP trials (1, 2, and 3), once-weekly semaglutide 2.4 mg produced substantial reductions in CRP. Studies suggest that semaglutide reduced hs-CRP in patients with T2DM comparative to placebo in adults with overweight or obesity [45,57]. In parallel, a recent meta-analysis of 13 randomized trials (>26,000 participants) showed that semaglutide consistently lowered CRP compared with placebo and other glucose-lowering therapies [58]. This positions GLP-1RAs as a potential strong multi-mechanistic treatment for inflammatory CVD.

Mechanistic work supports GLP-1RAs’ molecular anti-inflammatory and anti-atherosclerotic properties. In vitro GLP-1RAs blunt glucose-induced oxidative stress and nuclear factor-kappa B activation in endothelial cells, suppress expression of adhesion molecules, and reduce leukocyte extravasation. These effects are reproduced across rodent and primate models, with consistent reductions in IL-1β, TNF-α, and vascular inflammatory markers [59,60,61]. Small human studies further confirm reductions in oxidative stress biomarkers, such as plasma 8-iso-prostaglandin F2αlpha (plasma 8-iso-PGF2α) after dulaglutide treatment, adhesion molecules, and monocyte attractants [62,63,64]. GLP-1RAs promote cholesterol efflux, reduce triglyceride synthesis, and inhibit macrophage foam cell formation. GLP-1RAs also enhance endothelial nitric oxide synthase activity, suppress endothelin-1, and upregulate antioxidant defenses, while promoting anti-apoptotic and pro-angiogenic signaling [65,66,67,68]. These medications inhibit platelet aggregation by activating endothelial nitric oxide synthase signaling (eNOS) and cAMP, which reduces thrombus formation. These mechanisms have been observed to reduce early stroke formation. Additionally, GLP-1RAs moderate plaque formation via increased plaque collagen and promote M2 macrophage polarization, consistent with greater plaque stability and reduced risk of rupture [69,70].

In clinical studies, liraglutide reduced carotid intima-media thickness (CIMT) over 18 months in patients with T2DM and metabolic syndrome. A meta-analysis of 6675 patients found that exenatide was the most effective GLP-1RA in slowing CIMT progression among antidiabetic agents [71,72]. However, in a randomized placebo-controlled trial, exenatide did not significantly alter carotid plaque volume over 18 months, suggesting that GLP-1RAs may stabilize rather than reducing the size of established lesions [73].

In addition, GLP-1RAs modulate neuroinflammation. Experimental stroke models demonstrate that GLP-1R activation promotes microglial polarization toward the anti-inflammatory M2 phenotype, increases IL-4 and IL-10 secretion, and reduces pro-inflammatory cytokines, thereby limiting neuronal apoptosis and preserving peri-infarct tissue. Exendin-4 also preserves BBB permeability under ischemic stress, while morroniside and exenatide shift microglia from pro-inflammatory M1 states toward regenerative M2 profiles [74,75,76,77]. The balance between M1 and M2 phenotypes is a critical determinant of outcomes in stroke and other CNS disorders, including traumatic brain injury and Alzheimer’s disease [78,79].

Beyond inflammation, GLP-1RAs demonstrate direct anti-fibrotic activity. In cardiomyocytes, liraglutide suppressed glucose-induced collagen synthesis by blocking CD36-JNK-AP1 signaling [80]. In animal models, chronic GLP-1RA therapy prevented age-related cardiac fibrosis, attenuated extracellular matrix accumulation, and even reversed established myocardial remodeling. These findings suggest GLP-1RAs may protect the myocardium not only by reducing upstream stressors but by directly interfering with profibrotic pathways [81,82].

## 4. Clinical Data on Stroke Risk Reduction with GLP-1RAs

It is important to note that no randomized clinical trial (RCT) has yet evaluated stroke risk reduction with GLP-1RAs as an independent primary outcome, which is an important limitation when it comes to evaluating the effects of GLP-1RAs on stroke prevention. Nevertheless, several RCTs and meta-analyses of RCTs have suggested that GLP-1RAs are associated with decreased risk of stroke, particularly in patients with T2DM. A comprehensive meta-analysis of 28 RCTs involving 74,148 patients found that GLP-1 RAs were associated with a 17% reduction in adverse cerebrovascular outcomes (RR 0.83, 95% CI 0.76–0.91), with specific benefits for non-fatal stroke (RR 0.85, 95% CI 0.76–0.94) and ischemic stroke (RR 0.73, 95% CI 0.60–0.89) [83]. The benefits were most pronounced with longer-acting formulations, particularly dulaglutide and semaglutide, and appeared greater in patients with shorter diabetes duration and higher baseline eGFR. A 2024 meta-analysis of 11 cardiovascular outcome trials encompassing 82,140 participants confirmed a 15% relative reduction in total stroke (RR 0.85, 95% CI 0.77–0.93) and a 13% reduction in non-fatal stroke (RR 0.87, 95% CI 0.79–0.95) with GLP-1 RAs versus placebo [84]. The stroke reduction benefit was consistent across daily versus weekly formulations and oral versus subcutaneous routes, and it was present in patients both with and without diabetes. A pooled post hoc analysis of SUSTAIN 6 and PIONEER 6 cardiovascular outcome RCTs showed that semaglutide was associated with reduced incidence of any stroke by 32% (HR 0.68, 95% CI 0.46–1.00, *p* = 0.048), including a 49% reduced incidence rate in small-vessel occlusion strokes (HR 0.51, 95% CI 0.29–0.89) [12,85,86]. The benefit was consistent regardless of prior stroke history, though the effect was numerically stronger in patients without prior stroke (HR 0.60) compared with those with prior stroke (HR 0.89). Another major limitation in terms of interpreting data on stroke risk reduction with GLP-1RAs is the results from some trials that suggest no significant decrease in the incidence of stroke with GLP-1RAs compared with placebo. For instance, in the landmark SELECT trial, semaglutide was superior to placebo in reducing a composite of death from cardiovascular causes and non-fatal or non-fatal stroke [85,87]. However, when non-fatal stroke was analyzed as a separate outcome, similar event rates were observed in the semaglutide and placebo groups (1.7% of semaglutide-treated patients versus 1.9% of placebo patients (HR 0.93, 95% CI 0.74–1.15)), indicating that the observed cardiovascular benefit was driven primarily by reductions in non-fatal MI (HR 0.72) and cardiovascular death (HR 0.85). The lack of direct benefit on stroke risk reduction was replicated in the SOUL trial, which studied oral semaglutide in high-risk type 2 diabetes and demonstrated a 26% reduction in the primary composite major adverse cardiovascular event (MACE) endpoint (HR 0.74, 95% CI 0.65–0.85), with non-fatal stroke occurring in 3.0% of oral semaglutide patients versus 3.3% of placebo patients (HR 0.88, 95% CI 0.70–1.11) [88,89] While not statistically significant as an individual endpoint, these findings align with the overall cardiovascular benefit observed across the GLP-1 RA class, which could potentially be explained by the mechanisms described in Section 2 and Section 3. It is important to note that despite broadly favorable cerebrovascular effects observed across the drug class, not all agents have demonstrated comparable benefits. Notably, previously used agents such as albiglutide, lixisenatide, and extended-release exenatide failed to show significant stroke-protective effects, highlighting important heterogeneity and cautioning against assuming uniform stroke prevention across all GLP-1 receptor agonists (Table 1) [11,83,88,90,91,92,93,94,95,96,97,98,99,100,101,102]. A recent metanalysis of 85,373 patients comparing a GLP-1 receptor agonist for stroke prevention in patients with T2DM and without diabetes found an overall risk reduction of 15% of stroke (OR, 0.85, 95% CI 0.77–0.93) and 13% of non-fatal stroke (OR, 0.87, 95% CI: 0.79–0.95). However, the risk of fatal stroke was comparable in both groups (OR 0.94, 95% CI 0.75–1.17). Furthermore, pooled analysis regarding specific stroke subtypes suggested that GLP-1RAs did not significantly change the risk of hemorrhagic (OR 0.82, 95% CI 0.42–1.60), *p* = 0.57), ischemic (OR 0.85, 95% CI 0.64–1.13), *p* = 0.26), or embolic stroke (OR 2.33, 95% CI 0.49–11.09) compared to placebo. The analysis lacked specific data on the risk of stroke recurrence and overall mortality following a stroke [103]. RCT data evaluating stroke as an independent primary outcome for GLP-1RAs are lacking, which significantly limits the interpretation of existing trial findings in clinical practice. Further limitations are separately discussed in Section 7.2.

## 5. GLP-1 RAs and Cognitive Impact

At present, semaglutide is not approved by the U.S. Food and Drug Administration for the treatment of dementia or cognitive impairment. However, due to the increased interest in semaglutide therapy [104,105,106], recent real-world evidence has shown a promising connection with semaglutide use and lower risk of Alzheimer’s disease-related dementias (ADRD) in T2DM patients.

**Figure 2:**
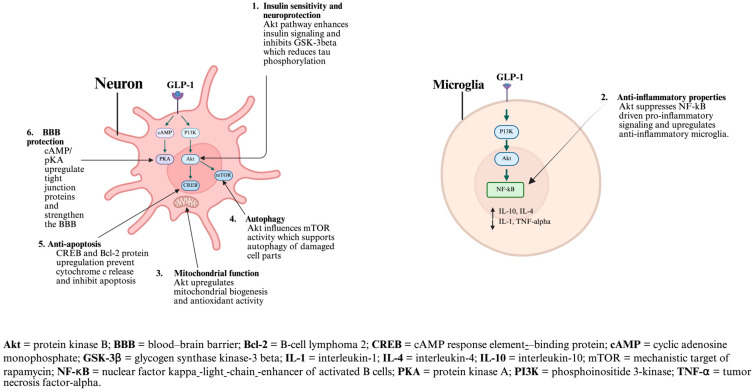
Neuroprotective and anti-inflammatory mechanisms of GLP-1 receptor activation in neurons and microglia [13,14]. Created in BioRender. Notani, S. (2026) https://BioRender.com/rg6y6lx (accessed on 20 March 2025).

T2DM is known to impair synaptic plasticity and neuronal survival; moreover, reduced insulin transport across the BBB leads to impaired neuronal insulin signaling and decreased cerebral glucose utilization [74,107,108]. GLP-1RAs act through a signaling cascade that typically involves the phosphoinositide 3-kinase/protein kinase B pathway, leading to inhibition of glycogen synthase kinase 3 beta (GSK-3 beta) with downregulation of tau phosphorylation, while the boost of cAMP acts on upregulating the tight junctions, consequently strengthening the BBB and improving the autophagy of cellular debris [109]. All together, these actions may potentiate the risk of dementia development (Figure 2). Mechanistic studies have also emphasized semaglutide as an anti-apoptotic agent, which reduces neuro-inflammation and increases synaptic function and neuronal survival [13,14,110,111].

Nationwide population-based databases were used in targeted trial emulations, which demonstrated no protective association in frontotemporal or Lewy body dementia. The strongest effect was in relation to vascular dementia, which was further supported by a pre-clinical systematic review carried out on animal models with cerebrovascular and neurodegenerative diseases [112,113,114]. Overall, semaglutide was associated with a statistically significantly reduced risk of ADRD incidence compared with metformin and compared with older-generation GLP-1RAs [114,115]. Interestingly, olfactory function improvement was reported, which may infer broader neuroprotective effects [111].

In the Liraglutide in Acute Minor Ischemic Stroke or High-Risk Transient Ischemic Attack with Type 2 Diabetes (LAMP) trial, a significant proportion of patients in the liraglutide group achieved excellent functional outcomes defined as an mRS < 1 at 90 days, which was better than in the control arm. Furthermore, the rates of symptomatic intracranial hemorrhage (ICH) and all-cause mortality were low and similar between the groups. In animal models, liraglutide has shown decreased infarct volume and improved neurological outcomes in middle cerebral artery occlusions [115].

Currently, two phase 3 clinical trials investigating semaglutide in people with early Alzheimer’s disease (EVOKE and EVOKE Plus) are evaluating once-daily semaglutide safety, tolerability, and efficacy in early Alzheimer’s disease compared to placebo groups [116].

## 6. Comparative Therapies with Other Antihyperglycemic Agents

GLP-1RAs have been compared to other major glucose-lowering therapies in practice regarding cardiovascular and cerebrovascular outcomes. Sodium–glucose cotransporter-2 (SGLT2) inhibitors, including empagliflozin, canagliflozin, and dapagliflozin, were not found to significantly reduce stroke risk compared to placebo or usual care. Large meta-analyses and collaborative trial data confirmed that SGLT2 inhibitors lower MACE through reductions in cardiovascular death and heart failure but have a net-neutral effect on AIS [117]. Direct head-to-head comparisons, such as target trial emulation studies, suggested that semaglutide may hold an advantage over empagliflozin for composite cardiovascular outcomes, including AIS, while dulaglutide did not show a similar benefit over empagliflozin [60,118]. The (SOUL) Semaglutide Cardiovascular Outcome trial also found that oral semaglutide reduced major adverse cardiovascular events independently of SGLT2 inhibitor use, with no safety concerns regarding combination therapy [89,119]. Meanwhile, dipeptidyl peptidase-4 (DPP-4) inhibitors are generally known to be cardiovascular-neutral for stroke risk; multiple Cardiovascular Outcome Trials (CVOTs) and meta-analyses have shown no significant reduction in MACE or AIS with DPP-4 inhibitors. (RR 0.96, 95% CI 0.85–1.08, I^2^ = 0%). That said, in certain patient populations, i.e., renal patients, they remain the preferred therapy [120,121].

It is important to note that insulin and sulfonylureas have not demonstrated stroke risk reduction in randomized trials. Both classes are considered cardiovascular-neutral, with no significant difference in AIS or MACE outcomes compared to other agents [122,123,124]. In addition, sulfonylureas carry a higher risk of hypoglycemia. Special consideration should also be given to patients with post-stroke autonomic dysfunction, who are especially vulnerable to swings in FBG [88,125]. Lastly, emerging incretin therapies, such as tirzepatide, are under investigation. Although there is no robust data on the stroke outcomes, the available data show potential [15,126,127]. Evidence suggests potential stroke risk reduction with tirzepatide (HR 0.71, 95% CI 0.62–0.81), though whether dual glucose-dependent insulinotropic polypeptide/GLP-1 receptor agonism confers additional cerebrovascular protection beyond selective GLP-1 receptor agonists remains uncertain [98]. Ongoing trials will further clarify their role in stroke prevention.

## 7. Conclusions

### 7.1. Safety and Tolerability Profile with Emphasis on Post-Stroke Care

Overall, GLP-1RAs portray a favorable safety profile; the main known side effects are gastrointestinal (e.g., nausea, vomiting, and diarrhea), which are typically transient and dose-dependent [128,129,130]. In regard to post-stroke care, the existing literature is limited yet favors the use of GLP-1RAs, since no risk of adverse events were found in the studied population and moreso showed potential benefits in reducing recurrent ischemic strokes (Figure 1). The Trial of Exenatide in Acute Ischaemic Stroke TEXAIS RCT, although aborted early due to COVID restrictions, showed participants treated with exenatide did not have a reduction in neurological impairment at 7 days in patients with AIS comparative to the placebo group (OR 1.22, 95% CI 0.79–1.88). However, exenatide did significantly reduce the frequency of hyperglycemic events and was safe to use. Adverse events of mild nausea and vomiting occurred in 3.5% of patients enrolled in the exenatide arm comparative to 0% in patients in the placebo group [131]. Their weight loss effects assist with rehabilitation; this further encourages GLP-1RA use in post-stroke rehab [132,133,134,135]. Some rare and serious events linked to GLP-1RAs are pancreatitis and gallbladder diseases; however, the evidence is inconclusive and should be monitored further. CVOTs did not show an elevation in stroke or neurological risks [125,126].

In the post-stroke setting, tolerability of the drug may be enhanced by starting at a lower dose and slowly titrating it, while paying attention to dysphagia and polypharmacy interactions. Nonetheless, the dehydration risk from GI side effects warrants hydration monitoring [15]. Drug adherence was another noted concern; where it was found that higher prescription co-payments were associated with lower 1-year adherence rates. Cost and access barriers contributed to underutilization, especially in low-income or uninsured cohorts, necessitating patient assistance programs or general formulations to improve equity [127,136,137].

GLP-1RAs are best understood as complementary to traditional prevention levers. As discussed in prior sections, GLP-1RAs may improve several upstream determinants of cerebrovascular risk including hyperglycemia, glycemic variability, obesity, blood pressure, inflammation, and endothelial dysfunction. The role of GLP-1RAs should be framed cautiously. Their emerging cerebrovascular benefit is biologically plausible and supported by meta-analyses, but at present they are best viewed as adjunctive therapies. Emerging stroke-specific data remains limited but provide early signals to support this framework. The 2025 LAMP trial demonstrated a significant reduction in recurrent ischemic stroke when patients were treated with liraglutide in addition to standard care [102], though similar trials with exenatide found no significant difference [131], highlighting ongoing uncertainty and heterogeneity within the drug class.

### 7.2. Limitations and Future Directions

Despite the presented data from CVOTs, several limitations exist. The use of GLP-1RAs for stroke risk reduction is limited by the fact that stroke was a secondary endpoint in CVOTs, which may reduce the statistical power for stroke-specific analyses, as well as by the relatively short duration of trials that restrict long-term outcome assessment. Overall, generalizability is limited, since patients with prior strokes are under-represented, and studies primarily enroll those with T2DM, excluding the broader populations such as non-diabetic patients or the elderly with a higher stroke risk [88,103,132,133].

Furthermore, the majority of trials had variable heterogeneity and did not include diverse populations in terms of race and ethnicity, which further limits generalizability. There was a lack of comparative evidence evaluating the effectiveness of different GLP-1RAs across stroke subtypes. Thus, more RCTs are needed to study the effects of GLP-1 RAs on stroke and cognitive function as independent primary outcomes. These studies should also include patients with prior stroke and non-diabetic high-risk patients based on emerging real-world evidence [16]. Elderly populations should also be included alongside comparison against other modifiable risk factors, i.e., lifestyle modifications. Both cardiology and neurology societies should collaborate to integrate cognition endpoints, given GLP-1 RAs’ potential neuroprotective effects, in addition to the amelioration of cognitive impairment and potential slowing of neurodegeneration [17,138,139,140].

### 7.3. Summary

In summary, GLP-1RAs provide promising but limited evidence for stroke risk reduction among glucose-lowering therapies. This narrative review aims to evaluate the current evidence on the neurovascular, cardiometabolic, and anti-inflammatory actions of GLP-1RAs and their potential relationship to stroke risk. However, as highlighted in the literature, in the majority of CVOT, stroke is usually a secondary outcome. Importantly, we acknowledge the paucity of randomized clinical trials specifically designed to evaluate stroke risk reduction with GLP-1RAs and the limited robust data on stroke prevention in non-diabetic populations. There exists a need for further research, including randomized control trials. With the widespread use of GLP-1 agonists, acknowledged in the media as the “Ozempic craze” [105,106,141], it feels imperative to close the knowledge gap for the general patient population. Future trials would benefit from performing head-to-head comparisons with other cardiometabolic therapies. In clinical practice, therapy choice should be individualized, considering comorbidities, patient reference, and the broader cardiovascular and renal benefits of each drug class.

## Figures and Tables

**Table 1:** Comparative clinical and mechanistic characteristics of GLP-1 receptor agonists relevant to stroke risk reduction [11,83,88,90,91,92,93,94,95,96,97,98,99,100,101,102].

Drug	Brand Name	Structural Notes	Major Elimination Pathway	Half Life	Adverse Cerebrovascular Risk Reduction in T2DM Population
Exenatide	Byetta (IR) Bydurean BCise (ER)	Based on exendin-4, from gila monster venom. 53% homology to Human GLP-1	proteolytic degradation, renal elimination. Dose adjustment required if eGFR < 45 mL/min/1.73 m^2^	2.4 h	Nonsignificant reduction in ACVE (HR: 0.85, 95%CI 0.70–1.03)
Liraglutide	Victoza, Saxenda	Native GLP-1 conjugated to palmitic acid, allows albumin binding and protects against DPP-4	DDP-4 proteolytic degradation. No dose adjustment necessary in CKD due to no renal or hepatic excretion	13 h	Nonsignificant reduction in ACVE (HR: 0.85, 95%CI 0.72–1.01)
Albiglutide	Tanzenum, Eperzan	GLP-1 dimer fused to human albumin	non-DDP-4 protein degradation with renal elimination, though no dose adjustment is indicated	5 days	Nonsignificant reduction in ACVE (HR: 0.86, 95%CI 0.65–1.12)
Dulaglutide	Trulicity	GLP-1 covalently linked to human IgG4-Fc	non-DDP-4 protein catabolism pathway. No dose adjustment indicated	112.8 h	Significant reduction in ACVE (HR: 0.78, 95%CI 0.63–0.95)
Lixisenatide	Adlyxin	Exendin-4 modified to confer DDP resistance	proteolytic degradation, renal elimination. Dose adjustment required if eGFR < 45 mL/min/1.73 m^2^	2–4 h	No effect on ACVE (HR: 1.15, 95%CI 0.82–1.61)
Semaglutide	Ozempic, Rybelsus, Wegovy	Human GLP-1 conjugated to fatty diacid, enables albumin binding and DDP-4 resistance	proteolytic degradation. No dose adjustment indicated	149–161 h	Significant reduction in ACVE (HR: 0.61, 95%CI 0.39–0.94) oral: significant reduction in ACVE (HR: 0.58, 95%CI 0.34–0.98)
Tirzepatide	Mounjaro, Zepbound	Based on native GIP conjugated to fatty diacid, enabled albumin binding and DDP-4 resistance	proteolytic degradation. No dose adjustment indicated	120 h	Significant decrease in stroke risk in T2DM (HR: 0.71, 95%CI 0.62–0.81)

## Data Availability

No new data was created or analyzed in this study. Data sharing is not applicable to this article.

## Abbreviations

The following abbreviations are used in this manuscript:

Abbreviation	Definition
ACC	American College of Cardiology
ACSL1	Acyl-CoA Synthetase Long Chain Family Member 1
ADRD	Alzheimer’s Disease-Related Dementia
AGE–RAGE	Advanced Glycation End Product–Receptor for Advanced Glycation End Product
AHA	American Heart Association
AIS	Acute Ischemic Stroke
Akt	Protein Kinase B
AMPK	Adenosine Monophosphate-Activated Protein Kinase
ASA	American Stroke Association
BBB	Blood–Brain Barrier
Bcl-2	B-Cell Lymphoma 2
BP	Blood Pressure
cAMP	Cyclic Adenosine Monophosphate
CIMT	Carotid Intima-Media Thickness
CNS	Central Nervous System
CREB	cAMP Response Element-Binding Protein
CRP	C-Reactive Protein
CVOT	Cardiovascular Outcome Trial
CVD	Cardiovascular Disease
DPP-4	Dipeptidyl Peptidase-4
eGFR	Estimated Glomerular Filtration Rate
eNOS	Endothelial Nitric Oxide Synthase
FBG	Fasting Blood Glucose
FDA	Food and Drug Administration
GLP-1	Glucagon-Like Peptide-1
GLP-1RA	Glucagon-Like Peptide-1 Receptor Agonist
GSK-3β	Glycogen Synthase Kinase-3 Beta
HbA1c	Hemoglobin A1c
HDL	High-Density Lipoprotein
hs-CRP	High-Sensitivity C-Reactive Protein
HR	Hazard Ratio
ICH	Intracranial Hemorrhage
IL-1	Interleukin-1
IL-4	Interleukin-4
IL-10	Interleukin-10
LDL-C	Low-Density Lipoprotein Cholesterol
MACE	Major Adverse Cardiovascular Events
MI	Myocardial Infarction
mRS	Modified Rankin Scale
mTOR	Mechanistic Target of Rapamycin
NF-κB	Nuclear Factor Kappa Light Chain Enhancer of Activated B Cells
NHE3	Sodium/Hydrogen Exchanger 3
OR	Odds Ratio
PI3K	Phosphoinositide 3-Kinase
PKA	Protein Kinase A
PPARγ	Peroxisome Proliferator-Activated Receptor Gamma
RCT	Randomized Controlled Trial
RR	Relative Risk
SANRA	Scale for the Assessment of Narrative Review Articles
SGLT2	Sodium–Glucose Cotransporter-2
T2DM	Type 2 Diabetes Mellitus
TIA	Transient Ischemic Attack
TNF-α	Tumor Necrosis Factor-Alpha
TOAST	Trial of ORG 10172 in Acute Stroke Treatment
